# Computational applications for the discovery of novel antiperovskites and chalcogenide perovskites: a review

**DOI:** 10.3389/fchem.2024.1468434

**Published:** 2024-10-11

**Authors:** Ming Sheng, Suqin Wang, Hui Zhu, Zhuang Liu, Guangtao Zhou

**Affiliations:** ^1^ College of Engineering, Shandong Xiehe University, Jinan, China; ^2^ Key Laboratory for Liquid-Solid Structural Evolution and Processing of Materials, Ministry of Education, Shandong University, Jinan, China

**Keywords:** perovskites, computational methods, antiperovskites, chalcogenide perovskites, stability, electronic structure

## Abstract

Novel perovskites pertain to newly discovered or less studied variants of the conventional perovskite structure, characterized by distinctive properties and potential for diverse applications such as ferroelectric, optoelectronic, and thermoelectric uses. In recent years, advancements in computational methods have markedly expedited the discovery and design of innovative perovskite materials, leading to numerous pertinent reports. However, there are few reviews that thoroughly elaborate the role of computational methods in studying novel perovskites, particularly for state-of-the-art perovskite categories. This review delves into the computational discovery of novel perovskite materials, with a particular focus on antiperovskites and chalcogenide perovskites. We begin with a discussion on the computational methods applied to evaluate the stability and electronic structure of materials. Next, we highlight how these methods expedite the discovery process, demonstrating how rational simulations contribute to researching novel perovskites with improved performance. Finally, we thoroughly discuss the remaining challenges and future outlooks in this research domain to encourage further investigation. We believe that this review will be highly beneficial both for newcomers to the field and for experienced researchers in computational science who are shifting their focus to novel perovskites.

## 1 Introduction

Perovskite and perovskite-related compounds represent an extensive class of materials with a multitude of applications across various domains of contemporary technology, including ceramics, thin films, solar cells, electronics, ferroelectrics, magnetic materials, superconductors, solid oxide fuel cells, ion conductors, catalysts, quantum materials, and biomedical applications (Dey et al., 2021; Jena et al., 2019; Kim et al., 2016; Zhang et al., 2016; Fu et al., 2019; Kovalenko et al., 2017; Wang K. et al., 2019). The unique structural characteristics of perovskites, primarily the ABX_3_ crystal structure, where A and B are cations of different sizes, and X is an anion, contribute to their diverse functionalities and tunable properties.

Novel perovskites refer to new or less explored variations of the traditional perovskite structure that exhibit unique properties and potential for various applications. These materials extend beyond the conventional ABX_3_ structure, incorporating modifications that provide enhanced or entirely new functionalities. Therefore, they display unique properties that broaden the functionalities of traditional perovskites, presenting promising opportunities for applications in energy, electronics, and other advanced technological industries.

The development of novel perovskite materials is a challenging process that demands significant time for theoretical design and experimental validation. Therefore, finding an effective toolbox to expedite the development process has become increasingly urgent. In recent years, advancements in computational methods have significantly accelerated the discovery and design of novel perovskite materials. Empirical factors such as the Goldschmidt tolerance (*τ*) factor and the octahedral index (*μ*) are essential for predicting the stability of perovskites based on ionic radii (Travis et al., 2016; Li et al., 2016). In addition, density functional theory (DFT) calculations offer a comprehensive understanding of the electronic, structural, thermodynamic, and mechanical properties of these materials, particularly when combined with high-throughput screening (HTS) techniques (Allam et al., 2018). In this case, HTS facilitates the rapid evaluation of numerous compositions to identify those with optimal properties for specific applications, including solar cells, light-emitting diodes (LEDs), and catalysis. Meanwhile, machine learning (ML) techniques improve the efficiency of exploring extensive chemical spaces by predicting material properties from large datasets, thereby reducing the computational costs associated with DFT calculations (Chen C. et al., 2020). Therefore, these approaches expedite the identification of novel perovskites with optimal properties, such as appropriate band gaps for solar cells or enhanced stability for prolonged applications.

Among these novel perovskites, antiperovskites and chalcogenide perovskites are especially noteworthy. Antiperovskites exhibit distinctive structural and electronic properties compared to their traditional perovskite counterparts. For example, molecular ferroelectrics with antiperovskite structure exhibit excellent ferroelectric properties (Shi et al., 2020a; Wei Z. et al., 2018; Ye et al., 2018; Liao et al., 2019; Zhang et al., 2018; Li et al., 2019), including higher Curie temperatures (*T*_C_), stronger ferroelectric polarization, larger piezoelectric coefficients, and lower coercive fields, which are superior to their counterparts with conventional perovskite structure (Wei W. et al., 2018; Tang et al., 2021; Chen XG. et al., 2020). The exploration of chalcogenide perovskites, another novel class of perovskites, has highlighted their potential in optoelectronic and thermoelectric applications due to their unique electronic structures and phase stability under various environmental conditions (Perera et al., 2016). Specifically, computational calculations reveal that predicted chalcogenide perovskites are ionic semiconductors with moderate band gaps. More importantly, most chalcogenide perovskites exhibit direct band gaps, which benefits to their optoelectronic properties. In addition, their diverse phase transitions and composition modifications with different chalcogenide elements (S or Se) result in the effective tuning of their band gaps. Therefore, chalcogenide perovskites behave potential for excellent optoelectronic properties. In addition to optoelectronic properties, Sb-based chalcogenide perovskites behave significantly enhanced thermal conductivity because of their p-type semiconducting feature. As a result, chalcogenide perovskites, with their ionic characteristics, exhibit exceptional thermoelectric potential.

**SCHEME 1:**
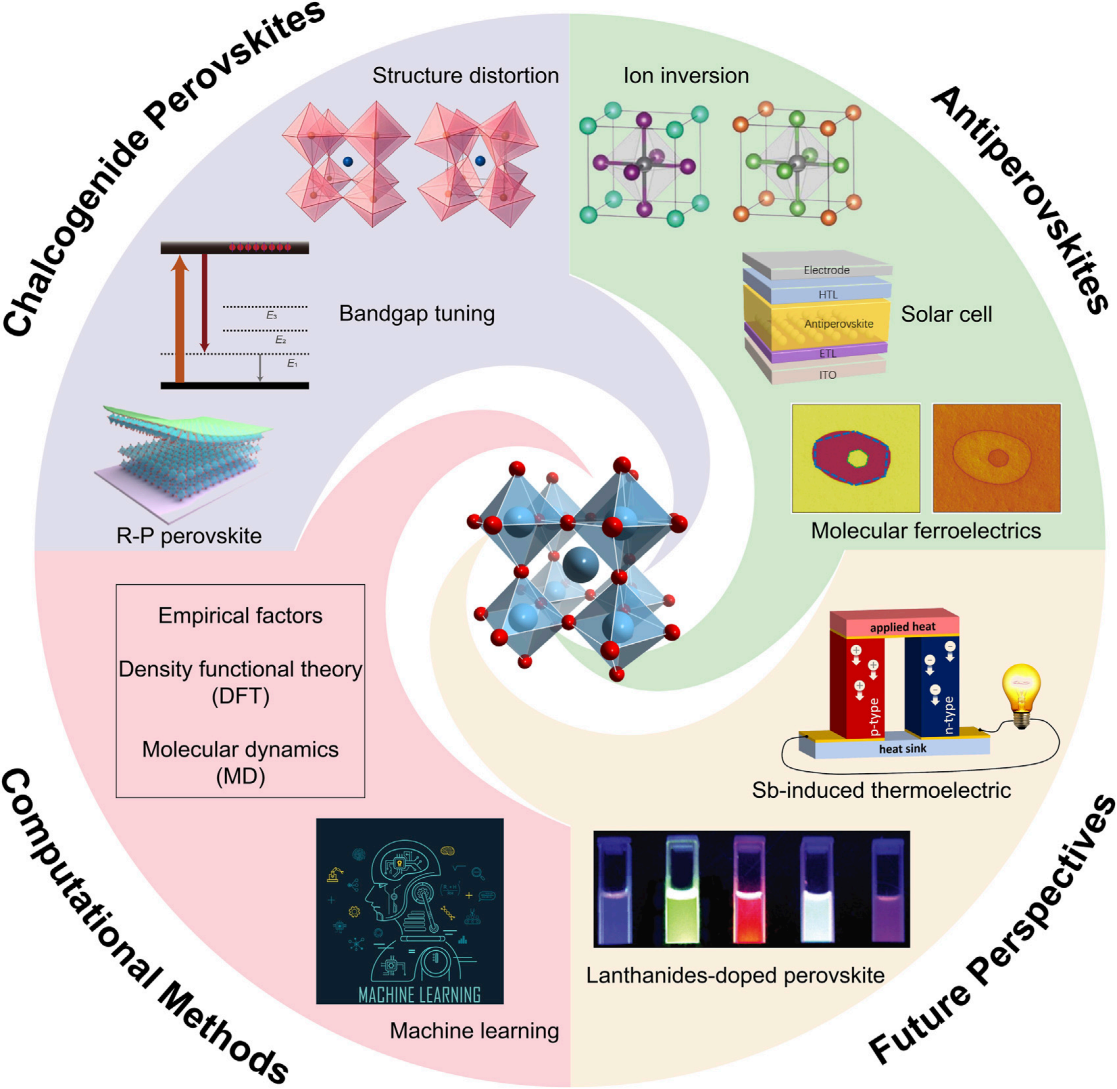
Computational applications on discovering novel antiperovskites and chalcogenide perovskites.

This review explores the computational discovery of novel perovskite materials, with focus on antiperovskites and chalcogenide perovskites, as shown in Scheme 1. It examines the computational methods used to assess the stability and electronic structures of these materials. Additionally, it highlights how these methods accelerate the discovery process, illustrating how rational simulations contribute to the research of novel perovskite materials with improved performance or unexpected phenomena. The integration of theoretical predictions with experimental validation emphasizes the potential of these advanced computational techniques in advancing the development of next-generation perovskite materials for various applications such as solid-state lighting, display, thermoelectric field, and so on.

## 2 Computational methods and applications

### 2.1 Computational methods

**SCHEME 2:**
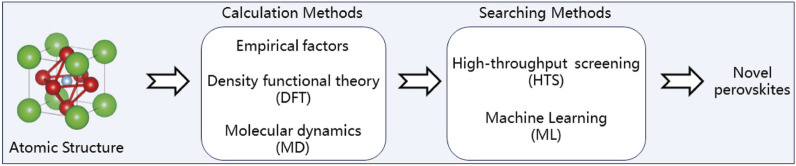
Accelerated discovery of novel perovskites via computational applications.

Following Scheme 2, the computational-enhanced discovery of novel perovskites begins with the consideration of empirical factors and computational methods, such as Density Functional Theory (DFT) and Molecular Dynamics (MD), to model and understand material properties at an atomic level. These computational approaches feed into high-throughput screening (HTS) processes, which allow for the rapid assessment of numerous material candidates. Finally, Machine Learning (ML) techniques are applied to analyze the data generated from HTS, enabling the identification of promising materials with desired properties more efficiently.

#### 2.1.1 Empirical factors

For perovskite materials, certain reliable empirical factors are used to assess structural stability. These factors enable a rapid evaluation of perovskite material stability through straightforward calculations, significantly reducing the required time compared to DFT simulations. Among these factors, ionic radii play a crucial role in shaping our understanding of bonding and stability in perovskites. For instance, Goldschmidt’s tolerance factor has been widely employed to assess the stability of perovskite structures (Travis et al., 2016; Li et al., 2016):

$$t=\left(r_{A}+r_{X}\right)/\sqrt{2}\left(r_{B}+r_{X}\right)$$

Here, *r*_i_ (where i denotes A, B, or X) represents the radius of a specific ion in perovskites. The calculated tolerance factors for typical perovskite materials range between 0.81 and 1.11. Therefore, predicting the stability of perovskites using the tolerance factor requires only their chemical compositions. Furthermore, when the octahedral index:

$$\mu =r_{B}/r_{X}$$

falls within the range of 0.41–0.90, it indicates significant distortion of the octahedral geometry and a high likelihood of multiphase coexistence (Travis et al., 2016). The ionic radii, tolerance factor, and octahedral factor are considered the most critical criteria for classification, highlighting that steric and geometric packing effects are essential to the stability of halide perovskites.

#### 2.1.2 Density functional theory

DFT calculation is a powerful computational tool for characterizing the electronic, structural, thermodynamic, and mechanical properties of perovskites, particularly when combined with high-throughput screening techniques (Allam et al., 2018). This approach facilitates a deeper understanding of perovskites and accelerates the discovery of novel materials with optimal properties. However, DFT derived from Kohn–Sham orbitals describing the conduction band minimum and maximum, approximate the real optical excitation problem but neglect excitonic effects. In practice, the selection of a proper DFT functional should be very careful (Vona et al., 2022).

#### 2.1.3 High-throughput screening

HTS involves the rapid and automated testing of large numbers of materials to identify those with desirable properties. It employs computational simulations and experimental techniques to evaluate electronic, optical, and structural properties across a wide range of compositions. This systematic approach accelerates the discovery and optimization of novel perovskite materials for various applications, including solar cells, LEDs, and catalysis.

#### 2.1.4 Global optimization technique

The actual structure of a material typically corresponds to the global minimum (GM) on the potential energy surface (PES), representing the lowest energy point. Identifying this GM is a crucial global optimization (GO) problem (23). Recently, advanced GO software such as CALYPSO, USPEX, and AIRSS have been emerged as powerful tools in the crystal prediction and discovery of novel structures, particularly for antiperovskites and chalcogenide perovskites. For example, Lee et al. successfully filtered 18,133 hypothetical X_3_BA down to seven exceptional candidates with outstanding room-temperature ionic conductivity, using GO techniques. Techniques such as genetic algorithms, basin hopping, minimum hopping, topological methods, particle swarm optimization, and tabu search have contributed to enhancing the efficiency and effectiveness of these optimization processes (Zhao et al., 2015).

#### 2.1.5 Machine learning technique

While accurate methods like DFT calculations have revolutionized material prediction and design, their high computational costs and limited scalability constrain their effectiveness in exploring the vast chemical spaces of novel perovskites (Chen C. et al., 2020). ML techniques facilitate the rapid and direct prediction of material properties from extensive datasets, significantly reducing the computational costs associated with DFT calculations and allowing for more efficient exploration of larger chemical spaces (Allam et al., 2018). Popular ML methods include graph neural networks, support vector machines (SVMs), and Gaussian processes (Pilania et al., 2016). Additionally, active learning combined with Bayesian optimization can efficiently identify novel perovskites from large materials databases. Table 1 presents a list of popular datasets used for screening novel perovskites. While some datasets include experimental data, the largest datasets typically rely on computational methods to generate labels. These datasets often focus on specific targets, such as the electronic bandgap, relevant for energy applications.

**TABLE 1:** Common datasets used for high-throughput screening and machine learning applications in the discovery of novel perovskites, with data sourced from experiments (*Exp.*) or calculations (*Cal.*). The amount of data is recorded as of August 2024, and can be still updated.

Datasets	Type	Description
Materials Project (Jain et al., 2013)	*Cal.*	An open-access databases provide material properties by predicting how both real and hypothetical new materials can be utilized.
Open Quantum Materials Database (OQMD) (Shen et al., 2022)	*Cal.*	DFT calculations determined the thermodynamic and structural properties of 1,226,781 materials.
Open Catalyst Project (Tran et al., 2023)	*Cal.*	Contains 1.3 million relaxed structures with results from over 260 million DFT calculation.
Automatic-FLOW for Materials Discovery (AFLOW) (Curtarolo et al., 2012)	*Cal.*	A globally accessible database has recorded 3,530,330 materials with various properties.
Cambridge Structural Database (CSD) (Allen, 2002)	*Exp.*	Contains over 1.25 million accurate 3D structures with data obtained from X-ray and neutron diffraction analyses.
The Inorganic Crystal Structure Database (ICSD) (Rühl, 2019)	*Exp.*	More than 210,000 characterization data points for inorganic crystal structures.
Crystallography Open Database (COD) (Gražulis et al., 2012)	*Exp.*	An open-access collection of crystal structures for organic, inorganic, metal-organic compounds, and minerals.

Note that the accuracy of DFT calculation is crucial. High-precision DFT ensures that the ML model learns from accurate representations of atomic structures and DFT-calculated result, which is essential for making correct predictions in new, unseen scenarios (Bogojeski et al., 2020).

#### 2.1.6 Molecular dynamics

To evaluate time- and temperature-dependent stability, molecular dynamics (MD) simulations can assess whether a perovskite maintains structural integrity under varying environmental conditions (Lu et al., 2018). Unlike static methods such as DFT calculations, MD focuses on the dynamic and statistical properties of a system (Shi et al., 2021). Additionally, solvent effects can be considered, as they can sometimes destabilize perovskites (Tan et al., 2020). Classical MD uses empirical force fields to model these interactions, which is computationally less intensive but may be less accurate for complex systems. In contrast, *ab initio* molecular dynamics (AIMD) employs quantum mechanical calculations to simulate atomic interactions, providing highly accurate results.

### 2.2 Applications of computational methods on evaluation of stability

Predicting the structural stability of perovskites remains a significant challenge in the discovery of novel perovskites. For new perovskite materials, the use of empirical factors to assess lattice stability may vary, necessitating adjustments for specific cases. For example, the effective tolerance factor (*t*_eff_) and octahedral index (*μ*_eff_) for quaternary antiperovskites X_6_B_2_AA′ and X_6_BB′A_2_ are defined as follows (Han et al., 2021):

$$t_{e\text{ff}}\;\left(X_{6}B_{2}{\text{AA}}^{\prime }\right)=\left(\frac{r_{A}+r_{A^{\prime }}}{2}+r_{x}\right)/\;\sqrt{2}\left(r_{B}+r_{x}\right)$$

$$\mu_{\text{eff}}\left(X_{6}B_{2}{\text{AA}}^{\prime }\right)=r_{B\;}/\;r_{x}$$

$$t_{e\text{ff}}\;\left(X_{6}{\text{BB}}^{\prime }A_{2}\right)=\left(r_{A}+r_{x}\right)\;/\;\sqrt{2}\left(\frac{r_{B}+{\;r}_{B^{\prime }}}{2}+r_{x}\right)$$

$$\mu_{\text{eff}}\;\left(X_{6}{\text{BB}}^{\prime }A_{2}\right)=\left(r_{B\;}+r_{B^{\prime }\;}\right)\;/\;2r_{x}$$

where *r*_i_ (i = A, A′, B, B′, X) stands for the radii of ions in antiperovskites.

Obtained from DFT calculations, the formation energy is crucial for assessing the stability and synthesizability of perovskites. A negative formation energy indicates that the material is energetically favorable to form and is likely to be stable under standard conditions relative to its constituent elements or compounds. Thermodynamic stability is typically estimated using either the DFT formation energy (*E*_f_, relative to its constituent elements) or the energy above the convex hull (*E*_hull_, relative to competing phases in the phase diagram) (Wang H. et al., 2020). Here, competing phases typically refer to the most stable phases among a range of conditions of temperature, pressure, or chemical composition. The formation energy can be used to identify the most energetically favorable structure, e.g., atomic orderings and types of novel perovskites (Jain et al., 2013; Togo and Tanaka, 2015). This energy is particularly significant when applied in HTS, such as in identifying stable M_3_XZ antiperovskites from the Materials Project database (Singh et al., 2018). However, since different compounds would form during the decomposition process under varying conditions (e.g., pressure and temperature), the convex hull will vary. Therefore, compared to *E*_hull_, *E*_f_ would be more practical in ML applications (Ong et al., 2008). Moreover, the incompleteness in the phase diagram or artificially stabilized phases from DFT errors can cause significant inaccuracies in *E*_hull_ (Chen C. et al., 2020). In contrast, *E*_f_ is a more reliable regression target.

In the study of novel perovskites such as antiperovskites and chalcogenide perovskites, structural distortion is of greater significance compared to traditional perovskites. These materials often display unconventional arrangements of A-site and B-site ions, resulting in pronounced structural distortions. For example, chalcogenide perovskites predominantly manifest three phases under ambient conditions compared to ideal cubic perovskite structure, namely, orthorhombic distorted phase, hexagonal phase, and needle-like phase (Tranchitella et al., 1997; Takeda et al., 1976; Lee et al., 2005; Lelieveld and IJdo, 1980). In some cases, the distorted phase exhibits better performances (Sun et al., 2015). Controlling and understanding these distortions is essential for tailoring functionalities. Furthermore, MD simulations conducted under various conditions, such as elevated temperatures, corroborate the robust stability of these hybrid chalcogenide perovskites (Baek et al., 2018; Li N. et al., 2020).

### 2.3 Applications of computational methods on electronic structure

The electronic structure is crucial in the research of novel perovskites such as antiperovskites and chalcogenide perovskites, as their unique electronic properties largely determine their performance. In antiperovskites, understanding the electronic structure helps elucidate their ferroelectric, topological, and transport properties, which are critical for applications in sensors, actuators, and quantum devices. Similarly, optimizing the electronic structure of chalcogenide perovskites can enhance their photovoltaic and thermoelectric performance.

The density of states (DOS) is pivotal in studying novel perovskites. The DOS at the Fermi energy significantly affects their magnetic, transport, thermoelectric, superconducting, and optoelectronic properties. The calculated electronic structure can be illustrated through a computed absorption spectrum, which corresponds to transitions between states with high DOS, providing detailed insights into how novel perovskites absorb light at different wavelengths (Kangsabanik et al., 2018; Ma et al., 2021). Dielectric properties, such as the dielectric constant and loss, impact the perovskites’ ability to store and release electric energy under an applied electric field. For novel perovskites, the dielectric response influences their suitability for various electronic applications, ranging from photovoltaics to electronic devices.

Among the various properties, the bandgap is a crucial parameter that significantly impacts performances. Tuning the bandgap is essential for optimizing the optoelectronic and thermoelectric properties of novel perovskites such as antiperovskites and chalcogenide perovskites. In photovoltaic applications, an optimal bandgap allows for efficient absorption of sunlight and conversion to electricity, maximizing the efficiency of solar cells. The Shockley-Queisser limit dictates that single-junction solar cells with an ideal bandgap of 1.34 eV can achieve a maximum solar conversion efficiency (SCE) of 33% (Rühle, 2016). Consequently, predicting or screening the bandgap is typically the initial step in computationally designing materials for solar cells (Chen C. et al., 2020). For thermoelectric applications, the bandgap influences the Seebeck coefficient and electrical conductivity, which are critical for effective thermal to electrical energy conversion. Additionally, the Seebeck coefficient and electrical conductivity are often calculated within the framework of Boltzmann transport theory using tools such as the BoltzTrap code (Schleder et al., 2019).

The Berry phase plays a significant role in the study of novel perovskites. For instance, it is used to calculate electric polarization in ferroelectric antiperovskites, aiding in determining the magnitude and direction of polarization in various states. Analyzing the change in polarization among different states, such as during a phase transition, can reveal the dynamics of polarization switching (Wang ZX. et al., 2019). Additionally, the Berry phase influences the transport properties of materials, such as the Hall effect and magnetoresistance, providing theoretical support for understanding conductivity and thermoelectric performance. By analyzing the Berry curvature, researchers can also identify and characterize topological phases in antiperovskites, such as topological insulators or Weyl semimetals (Kawakami et al., 2018).

The DFT electronic structure, derived from Kohn–Sham orbitals describing the conduction band minimum and maximum, approximate the real optical excitation problem but neglect excitonic effects; however, the accurate depiction of electronic structure is further complicated by the interplay between strong electron-electron interactions and spin-orbit coupling (SOC), which becomes important for heavy elements significantly reduce the band gap (Das et al., 2022). To address this, researchers often employ *ad hoc* DFT hybrid functionals tailored to match the band gap of each specific perovskite (IOPscience, 2011). While this approach introduces a degree of empiricism and limits the ability to reliably predict the properties and band gaps of unknown perovskites, it is also common practice to include SOC effects in calculations to improve accuracy (Umari et al., 2014). Through testing, G_0_W_0_ method performs best compared with experimental result but is computationally remarkably expensive. At the DFT level, HSE method gives overall the better estimation for novel perovskites (Vona et al., 2022; Fuchs et al., 2007). It is because that HSE incorporates a portion of exact exchange from Hartree-Fock theory, which helps improve the accuracy of the electronic structure calculations, particularly for systems with strong correlation effects (Janesko et al., 2009). Thus, HSE have currently widely utilized as a proper practice in the electronic structure calculation (Sun et al., 2015; Liu et al., 2021).

Some workflows were also developed to predict an accurate electronic structure. For example, Gebhardt et al. take the SOC, many-particle theory, and the dynamical behavior of the perovskites into account. The developed workflow could predict the band gaps within 0.2 eV as compared to experimental data. In some cases, the dynamical effects should be considered using the MD simulations, such for the higher symmetry of the α-phase (high-temperature) phase (Vona et al., 2022). Some workflows take the SOC, many-particle theory, and the dynamical behavior of the perovskites into account were also developed to predict an accurate electronic structure. For example, Gebhardt et al. (Gebhardt et al., 2021). developed such a workflow that comprises computations on an equal footing by efficient DFT methods and predict the band gaps within 0.2 eV as compared to experimental data.

It is worth noting that for novel perovskites, complex structural issues such as structural distortion, defects, impurities, and phase transitions under varying conditions can arise. These structural variations not only impact the perovskites’ physical properties but also induce changes in their electronic structure. For example, the electronic structure among different states of rotated BX_6_ octahedra reveals that it is significantly impacted by the distortion, due to the intricate hybridization from orbital interactions (Niu et al., 2018a).

## 3 Accelerated discovery of novel antiperovskites

As the most extensive family of perovskite materials, ABX_3_ -type compounds—where A and B are cations and X is typically oxygen, halide, or chalcogenide—have garnered significant global research interest due to their versatile multifunctionality and the intriguing scientific principles they embody (Protesescu et al., 2015; Nedelcu et al., 2015). A conventional perovskite structure consists of a framework of corner-sharing AX_6_ octahedra, with A cations occupying the interstitial sites (Varadwaj et al., 2022). In contrast to conventional perovskites, antiperovskites or so-called inverse perovskites are derivatives of the ideal perovskite structure in which the positions of cations and anions are reversed, resulting in an electronically inverted structure (Wang Y. et al., 2020; Lin et al., 2017; Zhong et al., 2023; Zhong et al., 2021; Han et al., 2022; Feng et al., 2023). Consequently, their fundamental structural unit is not a cation-centered octahedron BX_6_ but an anion-centered octahedron XA_6_. From a structural perspective, antiperovskites are expected to offer similar advantages to those of perovskite-type structures, as they can accommodate a wide range of elements, forming a diverse family of functional materials (Krivovichev, 2024). As anticipated, antiperovskites have exhibited a variety of intriguing properties, including ionic conductivity, superconductivity, negative thermal expansion, and the ability to serve as host materials for photoluminescence, among other functionalities. These properties are likely to drive a new phase of development for functional antiperovskites (Deng et al., 2022; Ohashi et al., 2020). Consequently, it is valuable to explore how computational methods can be used to strategically design antiperovskites to enhance their performance or uncover previously unforeseen phenomena.

In this section, we explore the prediction of three novel categories of antiperovskites using various simulation methods, offering a detailed explanation of each simulation process. Additionally, we compare the simulation results with corresponding experimental reports, demonstrating the good alignment of experimental results with theoretical calculations.

### 3.1 Quaternary antiperovskite materials

In a recent study, two novel classes of pnictogen-based quaternary antiperovskites, with the formulas X_6_B_2_AA′ and X_6_BB′A_2_, were devised through ion type inversion and anion ordering on perovskite lattice sites (Han et al., 2021). Their phase stability and tunable band gaps were comprehensively predicted using DFT calculations. Further screening of these materials for photovoltaic applications identified several stable compounds with suitable direct band gaps, small carrier effective masses, low exciton binding energies, and strong optical absorption due to dipoles, making them promising candidates for photovoltaic absorber materials. Notably, the theoretical maximum solar cell efficiencies for these stable antiperovskites exceed 31%, which is comparable to or even surpasses the efficiencies of MAPbI_3_ -based (MA = methylamine) solar cells (Han et al., 2021). This simulation study highlights the significant potential of quaternary antiperovskites in the optoelectronic field and provides a novel approach to designing lead-free and air-stable perovskite-based photovoltaic absorber materials.

**FIGURE 1:**
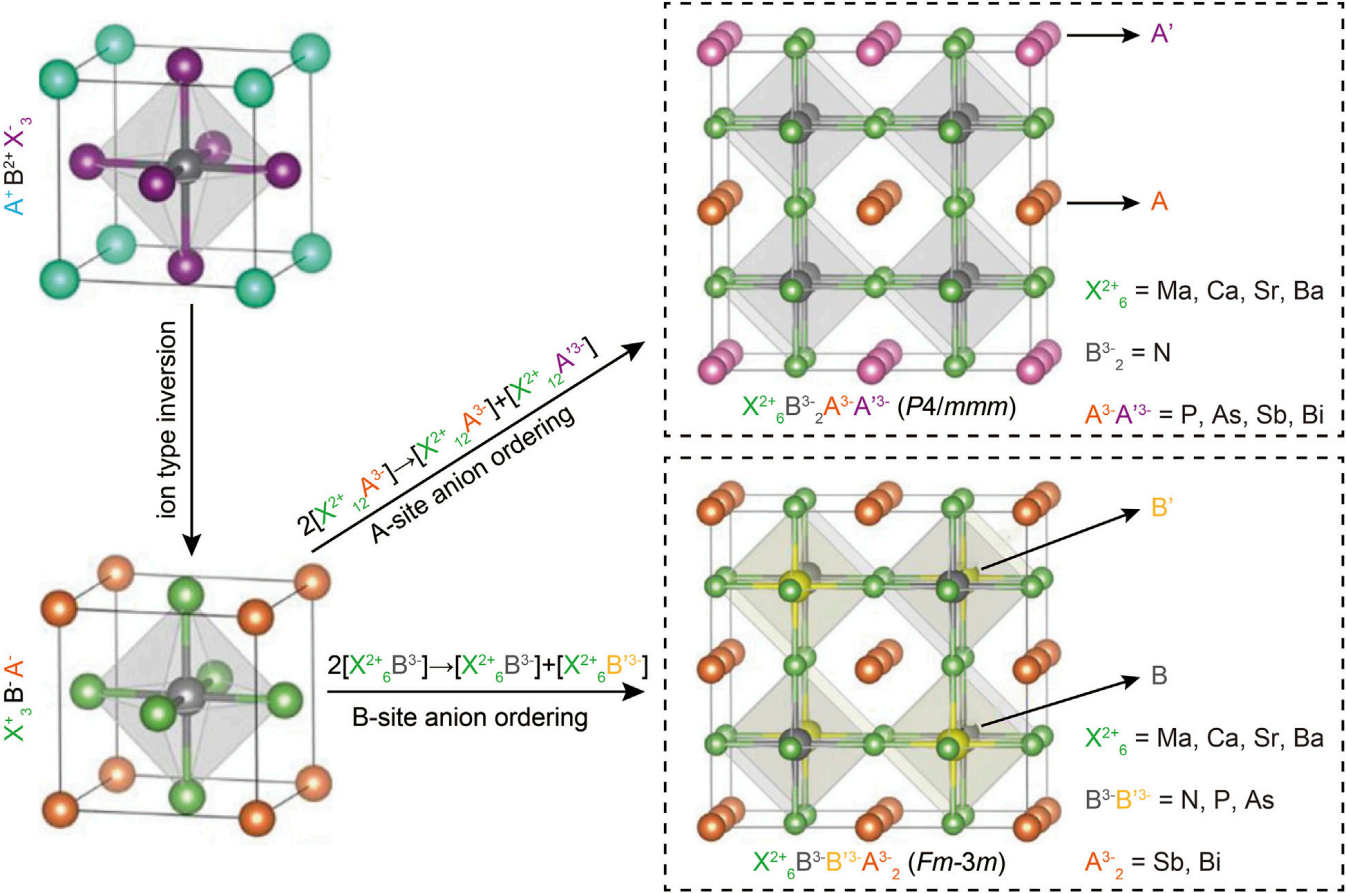
The schematic of two novel quaternary antiperovskites derived from typical cubic halide perovskites, along with the potential compositions. Reprinted with permission from Ref. (Han et al., 2021). Copyright 2021, American Chemical Society.

To predict the quaternary compositions and their corresponding lattice structures, DFT calculations were performed (Perdew et al., 1996; Kresse and Furthmüller, 1996). Unlike the typical perovskite configuration ABX_3_, antiperovskites X_3_BA feature an electronically inverted structure with one cationic X-site and two anionic A- and B-site, as illustrated in Figure 1. Building on previous reports that all-inorganic antiperovskites X_3_NA, where X represents divalent metals and A represents pnictogens, exhibit favorable optoelectronic properties for photovoltaic applications, the structure X_3_BAX was transformed into two novel quaternary antiperovskites: X_6_B_2_AA and X_6_BB′A_2_ through anion ordering. This process involves splitting the anionic A- or B-site, leading to a total of 48 potential component combinations, as shown in Figure 1.

**FIGURE 2:**
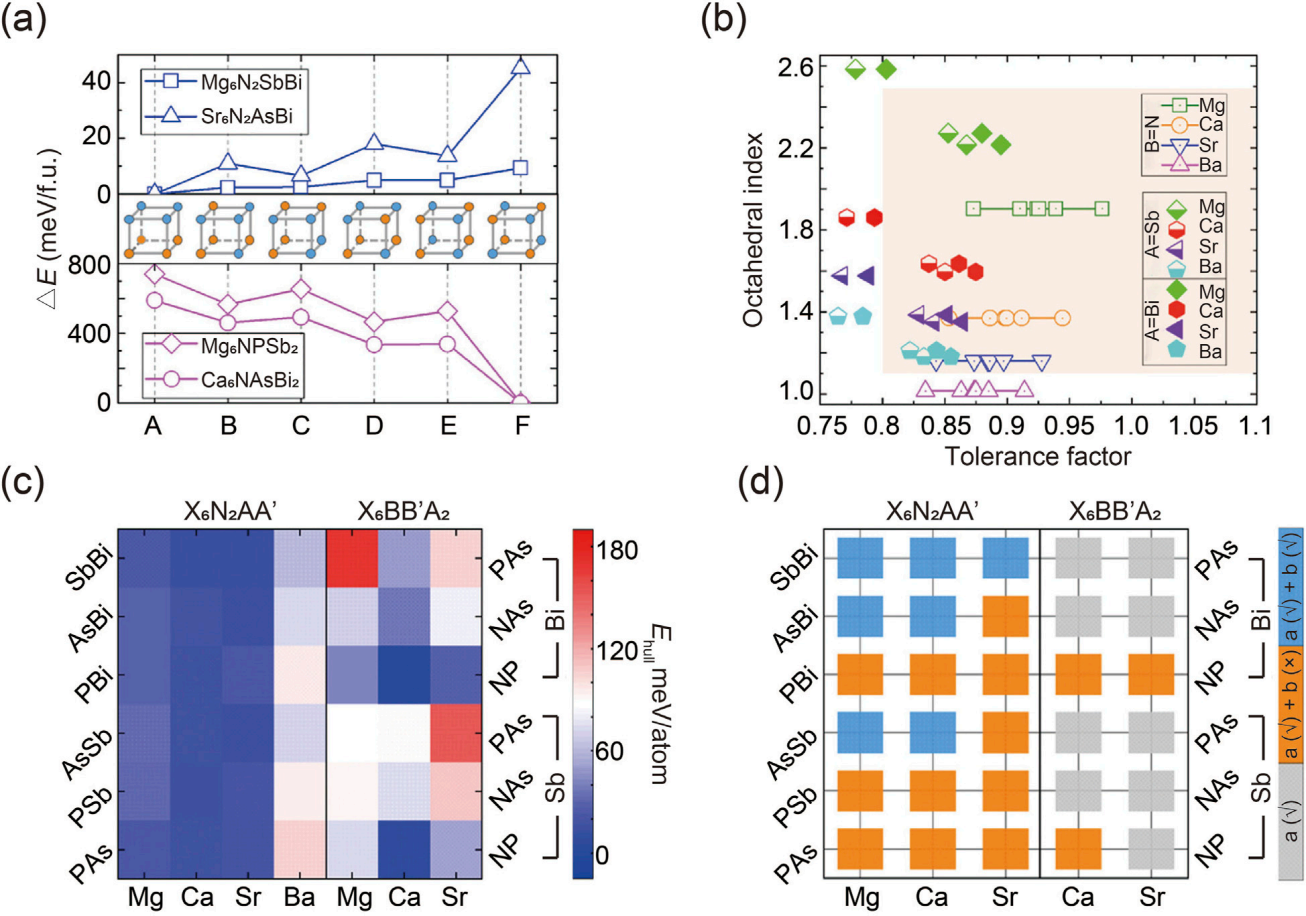
**(A)** Calculated total energies of two quaternary antiperovskites. **(B)** Statistical graph of potential quaternary antiperovskites involving tolerance factor and octahedral index. **(C)** Decomposition energy *E*_hull_ of two antiperovskites. **(D)** Evaluation of dynamic stability of thermodynamically stable antiperovskites. Gray squares represent compounds that are thermodynamically unstable, orange squares denote compounds that are thermodynamically stable but dynamically unstable, and blue squares indicate compounds that are stable both thermodynamically and dynamically. Reprinted with permission from Ref. (Han et al., 2021). Copyright 2021, American Chemical Society.

The prediction of potential antiperovskites began by identifying the most energetically favorable anionic ordering types in two structures (Jain et al., 2013; Togo and Tanaka, 2015). A supercell was constructed by doubling the standard cubic distortion-free unit cell of the antiperovskite X_3_BA. Various arrangements of AA′ and BB′ anionic pairs, denoted as A to F, were then considered within this supercell, as illustrated in Figure 2A. The calculated total energies of two A-site ordered quaternary antiperovskites (Mg_6_ N_2_SbBi and Sr_6_ N_2_ AsBi) and two B-site ordered quaternary antiperovskites (Mg_6_NPSb_2_ and Ca_6_ NAsBi_2_) were presented as examples. Simulation results show that the favored configurations of A- and B-site anion ordering in quaternary antiperovskites align with the cation ordering observed in conventional double perovskite oxides. Specifically, A-site cations tend to exhibit tetragonal layered ordering, while B-site cations prefer cubic rock-salt type ordering (King and Woodward, 2010). It is noteworthy that the simulated energy differences between various configurations of A-site cation-ordered regular double perovskites are typically minor, a finding consistent with previous reports (Gou et al., 2017).

To evaluate the lattice stability of the quaternary antiperovskites of interest, empirical metrics such as the tolerance factor and octahedral index were calculated (Travis et al., 2016; Goldschmidt, 1926; Kieslich et al., 2015; Kieslich et al., 2014). The results, shown in Figure 2B, indicate that most predicted compositions fall within the empirically stable region of traditional perovskites, while several unstable components were excluded.

Equally important as crystalline stability is thermodynamic robustness, which estimates the ease of decomposition for predicted compounds. Generally, a negative decomposition energy or *E*_hull_ indicates thermodynamic stability against degradation. Researchers integrated DFT calculations for specific quaternary compounds with data on all relevant elemental substances and binary and ternary phases from the Materials Project database. This approach expedited the assessment of thermodynamic stability, as illustrated in Figure 2C (Jong et al., 2016). By establishing a suitable threshold for *E*_hull_ (e.g., 25 meV/atom in this report, corresponding to *k*_B_T at room temperature), potential antiperovskites can be categorized as either thermodynamically stable or unstable, considering the potential for entropic stabilization (Dawson et al., 2018; Lu et al., 2020). Based on this criterion, several specific antiperovskites were identified as thermodynamically stable. Notably, compositions with large *E*_hull_ consistently exhibited crystallographic instability, suggesting a correlation between structural stability and thermal stability.

Furthermore, in addition to thermodynamic stability, dynamic stability—reflecting the gradual distortion of the structure over time—was assessed through phonon spectrum computations using the frozen phonon simulation technique (Togo and Tanaka, 2015). As shown in Figure 2D, seven quaternary antiperovskites are dynamically stable because they exhibit no imaginary modes (Han et al., 2021). Interestingly, simulation results reveal that antiperovskites with a higher degree of asymmetry demonstrate greater stability compared to typical cubic perovskites ABX_3_ and double perovskites A_2_ BB′X_6_. This finding is contrary to conventional expectations (Flerov et al., 1998; Glazer, 1972; Howard et al., 2003). As a result, distorted quaternary antiperovskites show promise for photovoltaic applications due to their enhanced dynamic stability and increased structural distortion, which can amplify the photoelectric effect through the boundary-breaking bulk photovoltaic effect.

### 3.2 Antiperovskite molecular ferroelectrics

Ferroelectrics are multifunctional electroactive materials with a wide range of applications (Liu et al., 2018a; Liu et al., 2018b). Their temperature-dependent spontaneous polarization can be modulated by electric fields or mechanical forces, making them highly suitable for temperature sensing, data storage, mechanical actuation, and energy harvesting (Liu et al., 2018c; Hussain et al., 2022; Liu et al., 2017). The phenomenon of ferroelectricity was first discovered in 1921 with Rochelle salt, followed by the identification of ferroelectric properties in other molecular systems. Significant progress in ferroelectric research was achieved with the discovery of ferroelectricity in perovskite materials like barium titanate (BTO) and lead zirconate titanate (PZT) (Lupascu and Rödel, 2005; Heywang, 1971; Luo et al., 2003; Vijatovic et al., 2008).

Unlike all-inorganic ferroelectrics, organic-inorganic hybrid ferroelectrics—often termed molecular ferroelectrics—offer several advantages, including lightweight construction, mechanical flexibility, and environmentally sustainable processing methods (Peng et al., 2020; Siwach et al., 2022; Liu et al., 2020). Recent studies have highlighted significant advancements in molecular ferroelectrics, characterized by high Curie temperatures, superior piezoelectric coefficients compared to all-inorganic ferroelectrics, sensitivity to polarized light due to chirality, photochromic properties, enhanced electrocaloric effects, and lanthanide-mediated fluorescence emission (Xu et al., 2020; Shi et al., 2020b; Xu et al., 2021; Xu et al., 2011; Fu et al., 2011; Tang et al., 2019; Zhang Y. et al., 2020; Shi et al., 2017).

**FIGURE 3:**
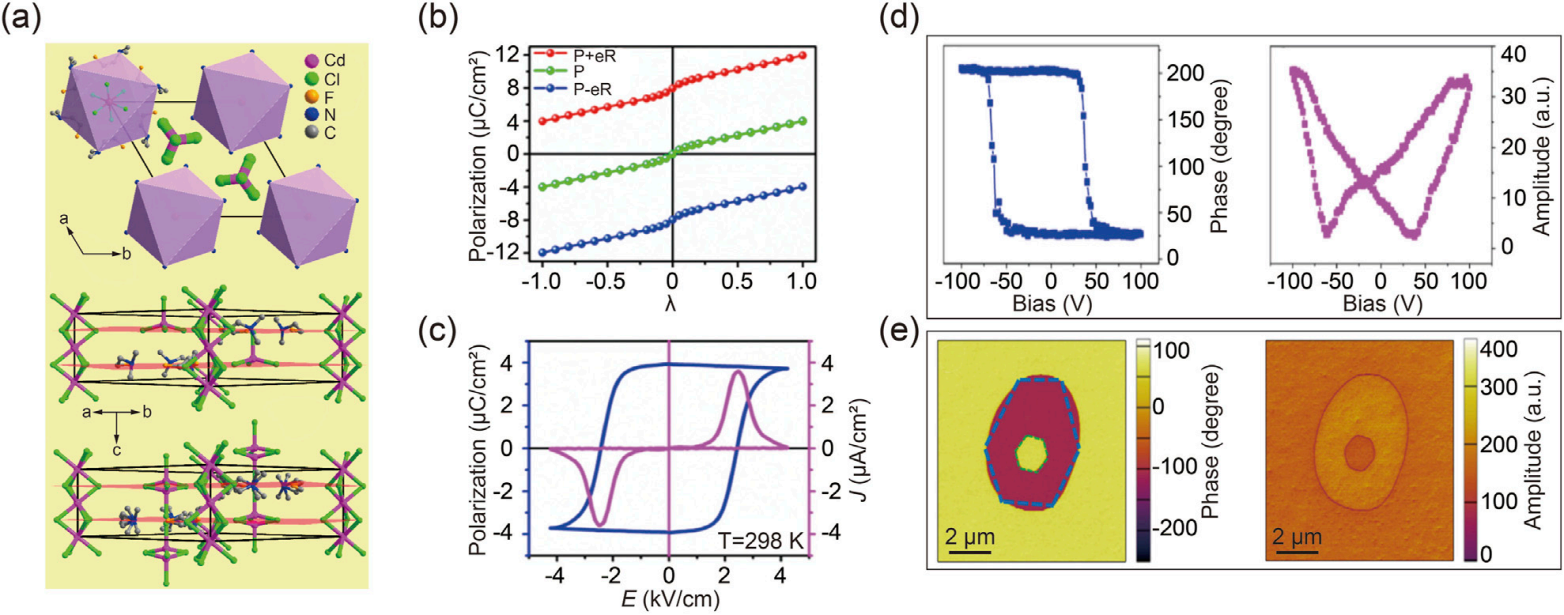
**(A)** The arrangement of designed antiperovskite molecular ferroelectrics [(CH_3_)_2_(F-CH_2_CH_2_) NH]_6_-CdCl_3_ (upper), along with its corresponding packing views in the ferroelectric (middle) and paraelectric (bottom) phases. **(B)** DFT calculated polarization strength as a function of structure distortion from nonpolar paraelectric phase to ferroelectric phases. **(C)** Spontaneous polarization strength as temperature varies, determined through the integration of pyroelectric current. **(D)** Typical phase and amplitude characteristics as a function of tip bias in molecular ferroelectrics. Reprinted with permission from Ref (Wei Z. et al., 2018). Copyright 2018, American Chemical Society. **(E)** Box-box switching of vertical phase (left) and amplitude (right) images. Reprinted with permission from Ref (Wang ZX. et al., 2019). Copyright 2019, American Chemical Society.

Since ferroelectric properties arise from the polarization asymmetry within the crystal lattice, DFT calculations can provide theoretical insights into the dynamics of polarization switching during the ferroelectric-paraelectric phase transition (Wang ZX. et al., 2019). The following example illustrates meticulously engineered antiperovskite molecular ferroelectric materials. Specifically, substituting one of the methyl groups in [(CH_3_)_3_NH]-CdCl_3_ with an ethyl group disrupted the existing mirror plane, potentially resulting in a polar crystal capable of displaying ferroelectric behavior. To further diminish the crystal symmetry, a fluorination strategy was employed to replace the ethyl group, thereby converting the perovskite structure into antiperovskites, as shown in Figure 3A (Zhang et al., 2020b; Shi et al., 2019). This compound demonstrates a ferroelectric phase below its *T*_C_, while a paraelectric phase emerges above the phase transition point (Wang ZX. et al., 2019).

The observed structural distortion in the ferroelectric phase accounts for both the organic cations and inorganic anions, considering the symmetry transition of the crystal structure. As illustrated in Figure 3B, DFT calculations define the paraelectric configuration as λ = 0 and the ferroelectric phase as λ = 1. Simulation results reveal that the polarization variation along the dynamic path shows that the positive extremum of ferroelectric polarization, initially along the c-axis from the starting state of λ = 1, gradually diminishes to zero at λ = 0 and then reverses to reach the end state of λ = −1 with negative extremum polarization [48]. This progression indicates a ferroelectric-paraelectric-ferroelectric phase transition, in alignment with previous studies on ferroelectric perovskites (Shi et al., 2020a; Wang S. et al., 2020; Pan et al., 2017). Subsequent experimental data have validated the DFT calculations’ accuracy, as depicted in Figure 3C. It is important to note that, according to recent polarization theory, quantum polarization might exaggerate the polarization strength, which should be excluded from calculations.

A fundamental characteristic of ferroelectrics is their electrically switchable spontaneous polarization. Therefore, a local Piezo-response Force Microscopy (PFM)-based hysteresis loop measurement was performed for antiperovskite molecular ferroelectrics, as shown in Figure 3D. The distinct 180° reversal of the PFM phase signal and the characteristic butterfly loop in the amplitude signal are indicative of polarization switching within ferroelectric domains (Fu et al., 2013; Zhang et al., 2013; You et al., 2017; Rabe et al., 2007; Resta, 1994; Spaldin, 2012). Furthermore, to further investigate dynamic ferroelectric switching in antiperovskite molecular ferroelectrics, an equal but opposite bias was applied to the materials. A box-in-box contrast image was then captured during both phase and amplitude transitions, as demonstrated in Figure 3E (Li L. et al., 2020; Zhang et al., 2020c; Wei et al., 2020).

In addition to demonstrating powerful capabilities in predicting compositions and functionalities for quaternary antiperovskites and antiperovskite molecular ferroelectrics, computational methods have also played a significant role in accelerating the development of compositions with advanced X-ray detection capabilities. In a very recent study, a novel antiperovskite (2-Habch)_3_Cl(PtI_6_) was developed that features indirect transition and low orbital symmetry (Liu et al., 2024). These characteristics enable such material to exhibit a carrier lifetime exceeding 3 ms, resulting in remarkable carrier mobility (*μτ* = 6.25 × 10^−3^ cm^2^ V^−1^) and high resistivity (10^12^ Ω cm). The electrical performance of this antiperovskite surpasses that of most existing X-ray detector materials. In-depth DFT calculations reveal that the low-dimensional characteristics of this material are the reason for the localized behavior of its band structure in various directions. Additionally, the low symmetry of the Pt 5d orbitals significantly reduces the dipole matrix elements. The indirect band gap nature of the material further decreases the dipole matrix elements.

In another groundbreaking study, researchers developed an atomic-position splitting method to design novel antiperovskites, effectively resolving the issue of suboptimal photovoltaic performance in previously reported antiperovskites (Tang et al., 2024). This research introduced a class of antiperovskite-derived materials with the formula X_3_BA_3_’, achieved by splitting the A anion, originally positioned at the corner site of the cubic antiperovskite structure, into three edge-centered sites, thereby preserving the three-dimensional octahedral framework. First-principles calculations confirmed that these potential compositions exhibit robust thermodynamic and dynamical stability. Notably, nine promising derivatives demonstrated maximum solar conversion efficiencies exceeding 24.5%, comparable to MAPbI_3_. Moreover, interpretable machine learning techniques were developed to identify critical regulatory factors that influence their thermodynamic stability and band gap. Therefore, computational methods play a crucial role in the design and development of novel semiconductor materials, guiding the optimization of their outstanding optoelectronic properties.

## 4 Accelerated discovery of chalcogenide perovskites

Over the past decade, there has been an unprecedented surge of interest in inorganic-organic hybrid perovskites, driven by their diverse chemical and physical properties (Egger et al., 2016). This intensified focus has led to significant breakthroughs, particularly highlighted by the dramatic increase in solar energy conversion efficiency from 3.8% to 25.5% within just 10 years (Kojima et al., 2009). Despite these advances, persistent challenges such as toxicity, degradability, and instability remain substantial barriers to their widespread industrial adoption. As a result, researchers are turning their attention to a new class of perovskites known as chalcogenide perovskites. Characterized by their lead-free composition, natural abundance in the Earth’s crust, and exceptional stability under ambient conditions, these perovskites offer a promising solution to the limitations of their predecessors (Swarnkar et al., 2019). Numerous experimental studies have successfully synthesized chalcogenide perovskites, revealing their potential and advantageous properties (Nie et al., 2018; Liang et al., 2023; Niu et al., 2018b; Niu et al., 2018c; Cao et al., 2014).

### 4.1 Typical chalcogenide perovskites ABX_3_

**FIGURE 4:**
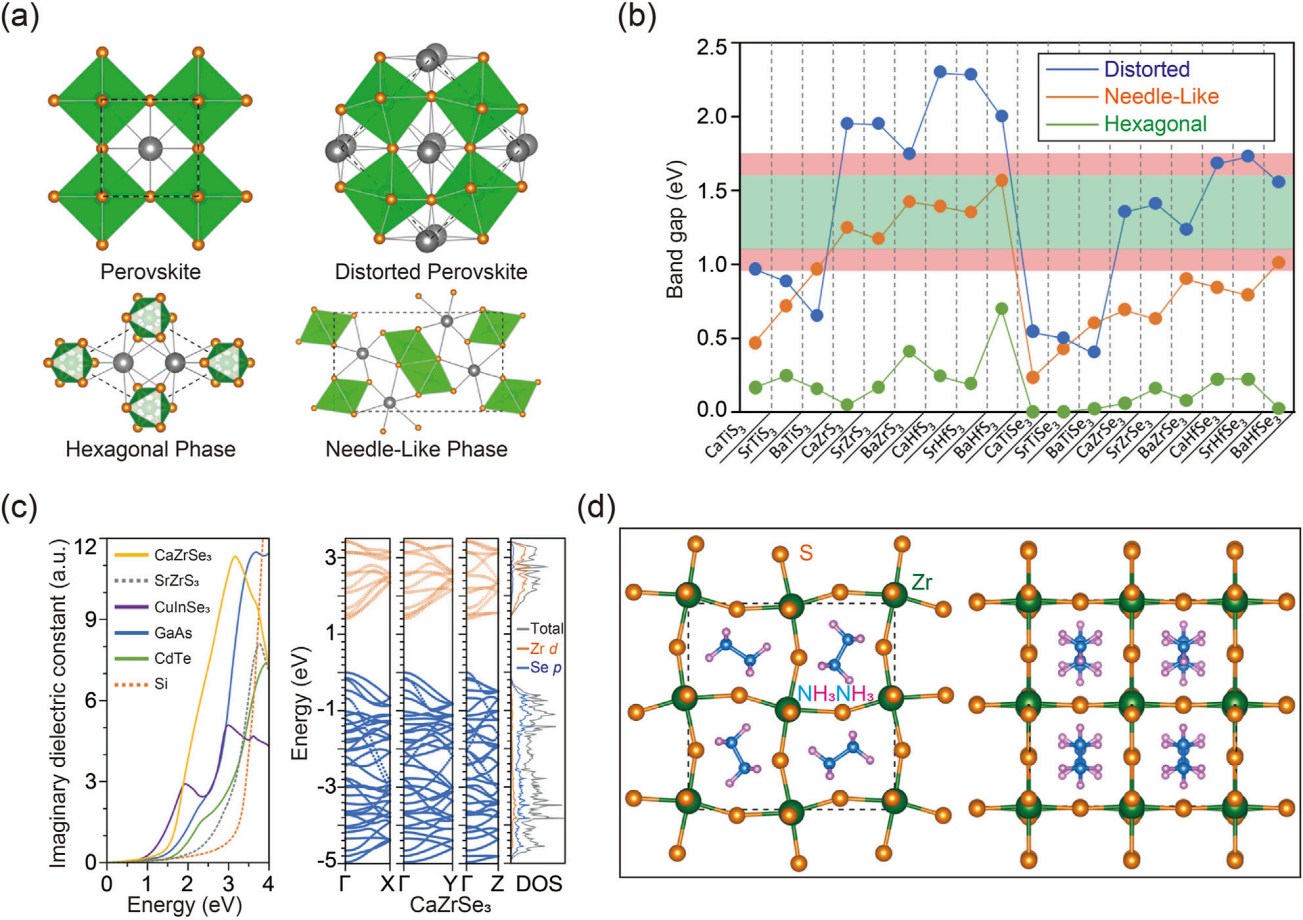
**(A)** Various phases for the ABX_3_ chalcogenide perovskites of cubic perovskite phase without distortion (upper left), distorted perovskite phase (upper right), hexagonal phase (bottom left), and needle-like phase (bottom right). **(B)** Calculated bandgaps of ABX_3_ chalcogenide perovskites obtained using DFT calculation. **(C)** Imaginary dielectric constant computed for CaZrSe_3_ with distorted perovskite phase and SrZrS_3_ with the needle-like phase according to DFT calculation (left). Band structure as well as the DOS of CaZrSe_3_ in the distorted perovskite phase (middle). Site-projected DOS alongside total DOS (right). **(D)** Atomic configuration of CH_3_NH_3_ZrS_3_ viewed along the c-axis (left) and perpendicular to the c-axis (right). Reprinted with permission from Ref (Sun et al., 2015). Copyright 2015, American Chemical Society.

Inspired by halide perovskites, chalcogenide perovskites are expected to display distinctive properties due to their ABX_3_ configuration, where A represents bivalent metals, B denotes tetravalent cations, and X stands for either sulfur (S) or selenium (Se) (Perera et al., 2016). As illustrated in Figure 4A, unlike the conventional perovskite structure, which is typically free of distortion, chalcogenide perovskites primarily exhibit three phases under ambient conditions, including orthorhombic distorted phase (No. 62, *Pnma*), hexagonal phase (No. 194, *P63/mmc*), and needle-like phase (No. 62, *Pnma*) (Tranchitella et al.; Takeda et al., 1976; Lee et al., 2005; Lelieveld and IJdo, 1980). Throughout these phase transitions, alterations in coordination numbers frequently occur. For instance, in the hexagonal phase, the cation coordination is analogous to that in the cubic perovskite structure, with A/B-site cations adopting a 12/6-coordination arrangement. Conversely, in the needle-like phase, the coordination number of the A cation is reduced to 9, signifying a collapse of the perovskite structure.

The band gaps of predicted chalcogenide perovskites were simulated using various calculation methods with carefully optimized parameters to ensure precision (Perera et al., 2016; Sun et al., 2015; Basera and Bhattacharya, 2022; Guo and Wang, 2019; Sun et al., 2019; Ju et al., 2017). As depicted in Figure 4B, simulation results reveal that, among the same chalcogenide perovskites, the distorted perovskite phase generally exhibits the largest band gap, while the hexagonal phase often shows the smallest band gap. Additionally, sulfide perovskites typically demonstrate larger band gaps compared to their selenide counterparts in both distorted and needle-like phases. This is attributed to the lower energy level of sulfur’s 3p orbitals compared to selenium’s 4p orbitals. Moreover, Hf-based chalcogenide perovskites usually exhibit larger band gaps compared to Zr-based counterparts, owing to the higher energy level of Hf 5d orbitals relative to Zr 4d orbitals. Importantly, the band gap calculations of potential chalcogenide perovskites have identified several promising candidates with suitable optical absorption ranges for solar cell applications. For example, some Zr- and Hf-based chalcogenide perovskites with needle-like or distorted phases and appropriate band gaps are suitable for solar cell fabrication, whereas their hexagonal counterparts, with relatively smaller band gaps, are less effective within the visible spectrum. Furthermore, the simulations confirm that several compositions with direct band gaps exhibit high power conversion efficiencies approaching the theoretical Shockley-Queisser limit, as indicated within the light red range in Figure 4B, highlighting their promising potential for photovoltaic applications (Shockley and Queisser, 1961).

The imaginary component of the dielectric function is a critical parameter for evaluating the optical absorption of semiconductor materials, with a higher dielectric constant generally indicating more intense absorption. Researchers have thus employed calculations to assess the absorption characteristics of chalcogenide perovskites, aiming to further evaluate their effectiveness for solar energy harvesting applications. Figure 4C displays the computational results of the dielectric function, compared to those of well-established solar cell materials (Burst et al., 2016; Adachi, 1985; Rockett and Birkmire, 1991; Ruch and Kino, 1968; Wasim, 1986). Among these compounds, CaZrSe_3_ in the distorted perovskite structure shows a dielectric function comparable to that of the commercial CuInSe_2_, suggesting its promising potential as a photovoltaic material. In contrast, although Zr- and Hf-based sulfide perovskites in the needle-like phase possess suitable band gaps, their optical absorption edges significantly surpass their fundamental band gaps. Consequently, chalcogenide perovskites in the needle-like phase exhibit indirect band gaps, making them unsuitable for solar cell applications. A deeper understanding of these distinctions can be obtained by analyzing their band structures using computational methods, as illustrated in Figure 4C. The lowest conduction bands display greater dispersion along the direction of the octahedral chains compared to the perpendicular directions, resulting in higher anisotropy of electron mobility in the hexagonal and needle-like phases compared to the distorted perovskite phase.

To identify additional potential chalcogenide perovskites, computational methods combined with stability assessments can be utilized through A- and B-site substitutions. It is noteworthy that substituting A-site constituents generally has a minimal impact on the overall properties of perovskite materials (Baek et al., 2018; Li N. et al., 2020). For example, inorganic A-site metals can be replaced with organic molecular cations, as demonstrated by the case of BaZrS_3_ depicted in Figure 4D. Computational results indicate that the band structure and properties of the optimized compounds closely mirror those of the original material. Additionally, AIMD simulations performed at elevated temperatures confirm the exceptional stability of these hybrid chalcogenide perovskites. These findings highlight the crucial role of computational methods not only in predicting material properties but also in expanding the range of possible material types.

### 4.2 Ruddlesden−Popper chalcogenide perovskites A_3_B_2_X_7_

Ruddlesden−Popper (R-P) phases, a specific structural type within perovskite materials, are characterized by the general formula A_2_' [A_n-1_B_n_X_3n+1_] and form two-dimensional (2D) geometrical configurations (Chen et al., 2018; Yu et al., 2017). These atomically thin layered structures exhibit notable octahedral rotations and distortions, which can lead to non-centrosymmetric configurations essential for polar characteristics and ferroelectric properties (Wang et al., 2016). Like conventional perovskite materials, chalcogenide perovskites can be engineered into two-dimensional R-P layered structures through compositional modifications, as indicated by simulation results. This approach enables the development of a wider variety of chalcogenide perovskite compounds.

**FIGURE 5:**
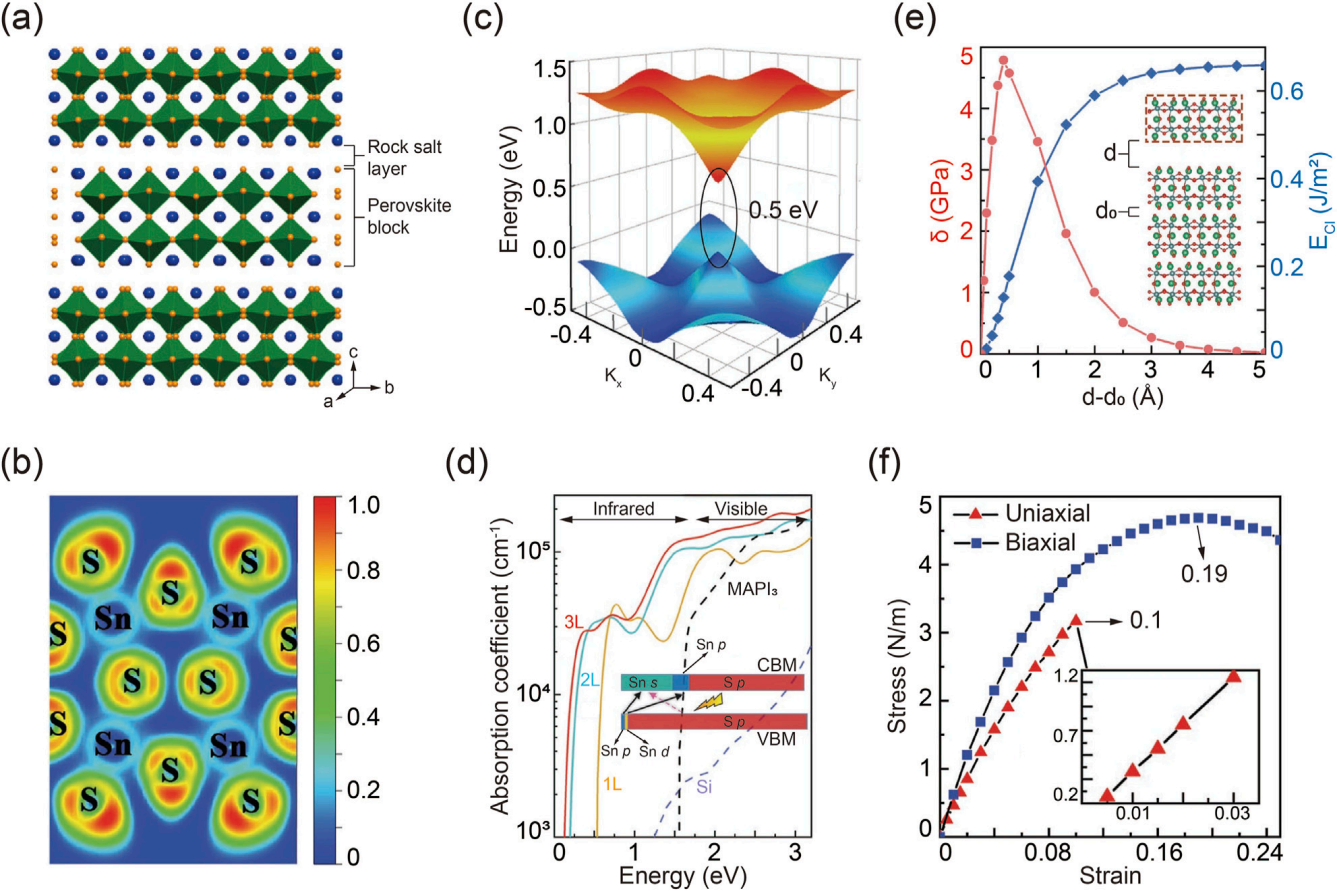
**(A)** Schematic packing configuration of R-P chalcogenide perovskites. Reprinted with permission from Ref (Niu et al., 2018a). Copyright 2018, American Chemical Society. **(B)** Simulated Sn-S bonding feature in R-P Ca_3_Sn_2_S_7_. **(C)** Three-dimensional band structure and corresponding bandgap of Ca_3_Sn_2_S_7_. **(D)** Calculated absorption behavior of R-P Ca_3_Sn_2_S_7_. **(E)** Calculated cleavage energy *E*_Cl_ and corresponding cleavage strength σ, varied by the distance of d-d_0_. **(F)** Stress evolution as a function of strain in R-P Ca_3_Sn_2_S_7_ under uniaxial and biaxial modes. Reprinted with permission from Ref (Du and Shi, 2019). Copyright 2019, Wiley.

The packing structure of R-P A_3_B_2_X_7_ chalcogenide perovskites is illustrated in Figure 5A, where two neighboring corner-sharing BX6 octahedra are separated by an intercalated AX layer (Niu et al., 2018a). To gain a deeper understanding of the bonding characteristics and stability of the 2D layered structure, an electron localization function (ELF) simulation was conducted using Ca_3_Sn_2_S_7_ as an example, as shown in Figure 5B (Du and Shi, 2019). An ELF value approaching zero indicates a metal-chalcogenide ionic bond with minimal electron density between the ions. Conversely, an ELF value approaching one signifies a strong covalent bond. Notably, the simulation reveals a partial covalent bond with an ELF value approximately 0.25 between Sn^4+^ and S^2-^, which contrasts with the ionic bonds typically found in halide perovskites (Cai et al., 2018). This Sn-S covalent bonding enhances the structural stability and charge transfer efficiency in 2D R-P chalcogenide perovskites. Additionally, compared to halide perovskites, the structural integrity of R-P chalcogenide perovskites is further reinforced by stronger Coulomb interactions among ions with higher valence states.

Simulation methods were employed to investigate the band structure of R-P chalcogenide perovskites. It is important to note that standard DFT calculations tend to underestimate band gaps. Therefore, the modified GGA-1/2 method proved more accurate for determining the true band gap. The three-dimensional band structure, illustrated in Figure 5C, highlights the band edge dispersion features of R-P chalcogenide perovskites. Remarkably, these materials exhibit semiconductor properties like graphene, characterized by linear electronic band edge dispersion and a relatively small direct quasi-particle band gap. Further band structure calculations under various configurations of rotated octahedra show that the electronic structure is notably influenced by the distortion of the BX_6_ octahedra, due to the complex hybridization from the interaction between the delocalized p orbitals of S and the s orbitals of Sn atoms (Niu et al., 2018a). According to the optimized band structure, R-P chalcogenide perovskites demonstrate intriguing optical absorption characteristics, as depicted in Figure 5D (Ravindran et al., 1997). They exhibit a broad absorption spectrum extending from the infrared to the ultraviolet regions, with absorption intensity increasing by two orders of magnitude in the infrared range, attributed to their direct band gap. Notably, the absorption edge shifts to higher energies with a decrease in layer number, due to the quantum confinement effect. Furthermore, the optical absorption capacity and spectral width of R-P chalcogenide perovskites surpass those of other advanced photovoltaic materials, such as MAPbI_3_ and silicon, highlighting their promising potential for solar cell applications (Wu et al., 2017; Alsalloum et al., 2020).

Mechanical exfoliation techniques are commonly used to obtain 2D monolayer materials from their 3D counterparts, which requires that the layered materials exhibit weak interlayer interactions (Liao et al., 2018; Huang et al., 2020; Yi and Shen, 2015). R-P chalcogenide perovskites, being a type of layered material, are amenable to easy exfoliation. The calculated cleavage energy *E*_Cl_, defined as the minimum energy required to overcome interlayer coupling during the exfoliation process, is depicted in Figure 5E (Medvedeva et al., 1996; Zhao et al., 2014). As the separation distance increases, *E*_Cl_ reaches its maximum value but remains significantly lower compared to other layered materials such as Ca_2_N (Druffel et al., 2016). Similarly, the cleavage strength σ shows a comparable trend.

Beyond exfoliation characteristics, mechanical strength and elastic strain limits are crucial, determined by analyzing the stress-strain relationships of layered chalcogenide perovskites. The Young’s modulus, obtained from the stress-strain curve using linear regression, is shown in Figure 5F. Notably, the stress-strain curves along the *x*-direction and *y*-direction are identical, indicating that 2D layered chalcogenide perovskites are isotropic elastic materials. As tensile strain increases, these perovskites approach their breaking limit, with an elastic strain limit of approximately 20%, comparable to layered MoS_2_ and graphene (Andrew et al., 2012; Mortazavi and Cuniberti, 2014).

In addition to optical and mechanical properties, DFT calculations predict the potential for ferroelectricity in R-P layered chalcogenide perovskites. Simulation results demonstrate that ferroelectric chalcogenide perovskites A_3_B_2_X_7_ can transform from their paraelectric ABX_3_ counterparts (Wang et al., 2016). Theoretical analysis highlights the tolerance factor as a key determinant of ferroelectric behavior (Zhang et al., 2017). For example, both Ba_3_Zr_2_S_7_ and Ba_3_Hf_2_S_7_, which have large tolerance factors, remain paraelectric due to the suppression of in-phase rotation—an essential feature for hybrid improper ferroelectricity. In contrast, Ca_3_Zr_2_S_7_, Ca_3_Hf_2_S_7_, and Ca_3_Zr_2_Se_7_, with smaller tolerance factors, exhibit in-phase rotation and can stabilize into the ferroelectric phase with significant polarization.

### 4.3 Other potential chalcogenide perovskites

In addition to ABX_3_- and A_3_B_2_X_7_-type chalcogenide perovskites, computational methods have predicted the potential of other chalcogenide perovskite variants, including NaBiX_2_, A′La_2_BX_10_ (where A′ = Ba, Sr, Ca; B = Hf, Zr), CsLnMX_3_ (where Ln = rare earth elements; M = Zn, Cd, Hg), and A_2_MM′X_6_ (where M = Bi or Sb; M′ = V, Nb, Ta), which are in their early research stage. Each of these materials offers unique advantages. For example, NaBiS_2_ exhibits higher absorption coefficients compared to established direct-bandgap thin-film absorbers and has potential as a photocatalyst for degrading organic matter (Huang et al., 2022; Rosales et al., 2018; Kumar et al., 2022). Additionally, A’La_2_BX_10_ shows higher carrier mobility, stronger optical absorption intensity, and an optimal Shockley-Queisser limit, making it a promising candidate for solar cell materials (lang et al., 2020). Lanthanides in CsLnMX_3_ can display characteristic emission through intrinsic 4f-4f transitions (Park et al., 2022; Yao et al., 2004; Mitchell et al., 2003; Jin et al., 2007). Furthermore, Sb-doped SrHfSe_3_ chalcogenide perovskites are predicted to possess thermoelectric properties due to their p-type semiconducting nature (Moroz et al., 2018).

## 5 Outstanding challenges and future perspectives

The exploration of novel perovskites, including antiperovskites and chalcogenide perovskites, represents a significant advancement in materials science. These materials offer unique properties that extend the functionalities of traditional perovskites, presenting promising opportunities for applications in energy, electronics, and other high-tech industries. In this review, we highlight the crucial role of advanced computational methods in the discovery and optimization of these novel perovskites, particularly focusing on antiperovskites and chalcogenide perovskites. We elaborate the computational techniques used to assess the stability and electronic structure of these materials, which accelerate the discovery process of new perovskites. Overall, the integration of advanced computational methods with experimental approaches is driving the development of the next-generation of high-performance perovskite materials.

Although the application of computational methods in discovering novel perovskites has advanced significantly in recent years, there are still many key challenges and opportunities within this emerging field, especially for ML techniques.

### 5.1 Interpretability of machine learning techniques

ML techniques have significantly accelerated the identification of promising materials by efficiently exploring vast chemical spaces. However, a key challenge is developing ML techniques that are not only accurate but also interpretable. Scientific disciplines often prefer simpler, more interpretable models over complex “black box” approaches. To enhance model interpretability, several strategies can be employed:

(1) Graph-Based Models. These models encode bonding relationships between atoms in crystals, creating frameworks that naturally incorporate physical and chemical constraints. This approach helps make the models more interpretable by aligning with known chemical principles.
(2) Symbolic Regression. This technique generates mathematical formulas or visual representations that illustrate how input features influence model predictions. By revealing the underlying relationships between features and predictions, symbolic regression helps demystify the “black box” nature of deep learning models.

### 5.2 Data challenges and solutions

Another major challenge in applying ML to materials science is the limited availability of high-quality data, particularly for specific and difficult-to-measure properties. While datasets for general properties like formation energies and band gaps are relatively abundant, niche properties such as alkali ionic conductivities or surface binding energies often suffer from data scarcity. To address these challenges, several strategies can be employed.

(1) Transfer Learning. This approach leverages knowledge from well-established models in domains with ample data (e.g., formation energies) to enhance model performance in areas with limited data (e.g., elastic moduli). By transferring insights from one domain to another, transfer learning can help mitigate data scarcity issues and improve predictive accuracy.
(2) Data Fusion: Combining data from various sources or types, such as integrating computational data with experimental results, can enhance the quality of overall dataset and provide a more comprehensive basis for model training.

By employing strategies, researchers can address the data limitations in the discovery of novel perovskites and improve the effectiveness of ML models.

Although aforementioned challenges related to small data, transfer learning, and data fusion have been well-established in materials science field, they are particularly pertinent in this emerging field where the exploration of novel perovskites often outpaces the availability of high-quality data. Our discussion goes beyond simply acknowledging these challenges by highlighting recent advancements and specific strategies tailored to overcome data limitations in the context of novel perovskites. For instance, we explore how graph-based models and symbolic regression can enhance the interpretability of ML techniques in these materials, making the “black box” nature of models more transparent. Such techniques are particularly usefully in crystals system like perovskites.

## 6 Conclusion

The exploration of novel perovskites, such as antiperovskites and chalcogenide perovskites, represents a significant advancement in materials science. These materials exhibit unique properties that extend the functionalities of traditional perovskites, offering promising opportunities for applications in energy, electronics, and other high-tech industries. In this review, the pivotal role of advanced computational methods in the discovery and optimization of novel perovskite have been highlighted, particularly antiperovskites and chalcogenide perovskites. It discusses the computational methods employed to evaluate the stability and electronic properties of these materials, thus, to accelerated discovery process of novel perovskites. Overall, the combination of advanced computational methods with experimental work is paving the way for the development of the next-generation of high-performance novel perovskite.
